# An Argument for Compulsory Vaccination: The Taxation Analogy

**DOI:** 10.1111/japp.12400

**Published:** 2019-11-01

**Authors:** Alberto Giubilini

**Affiliations:** ^1^ Oxford Martin School & Wellcome Centre for Ethics and the Humanities University of Oxford Oxford UK

## Abstract

I argue that there are significant moral reasons in addition to harm prevention for making vaccination against certain common infectious diseases compulsory. My argument is based on an analogy between vaccine refusal and tax evasion. First, I discuss some of the arguments for compulsory vaccination that are based on considerations of the risk of harm that the non‐vaccinated would pose on others; I will suggest that the strength of such arguments is contingent upon circumstances and that in order to provide the strongest defence possible of compulsory vaccination, such arguments need to be supplemented by additional arguments. I will then offer my additional argument for compulsory vaccination: I will argue that in both cases of vaccine refusal and of tax evasion individuals fail to make their fair contribution to important social and public goods, regardless of whether each individual contribution ‘makes a difference’. While fairness considerations have sometimes been used to support a moral duty to vaccinate, they have not been appealed to in order to argue for a legal duty to vaccinate. I will suggest that this is due, among other things, to a misapplication of the principle of the least restrictive alternative in public health. Finally, I will address nine possible objections to my argument.

## Introduction

Many countries have recommended coercive vaccination schedules, which typically include the pneumococcal vaccine, the MMR (measles‐mumps‐rubella) vaccine, the ‘6 in 1’ vaccine, and many others, administered at different ages, mostly during childhood. These policies aim to protect both those who will receive the vaccination and, through herd immunity, those who for some reason cannot be vaccinated – for example, they are immunosuppressed or allergic to vaccines or are too young to receive certain vaccines – as well as those for whom vaccination turns out to be ineffective. There is herd immunity when a sufficiently large number of individuals in a certain community is vaccinated against a certain infectious disease, so that the non‐vaccinated are also protected from the disease in virtue of a very low probability of an outbreak.^1^ The threshold for herd immunity varies depending on the disease in question; for example, herd immunity requires a 95% immunisation rate for measles and 92–94% for pertussis.

Vaccination policies are coercive when they attach significant enough penalties to non‐vaccination.^2^ Both mandatory and compulsory vaccination are instances of coercive policies. Although the two terms are often conflated, Mark Navin and Mark Largent^3^ usefully distinguish between ‘mandatory’ vaccination and ‘compulsory’ vaccination. According to their characterisation, mandatory vaccination is the withholding of valuable social goods or services from families who choose not to vaccinate their children for non‐medical reasons; for example, in the United States, vaccination is required for enrolment in public schools (although most states allow ‘conscientious objection’), and in Australia, the state withholds certain financial benefits from families that do not vaccinate their children. ‘Compulsory vaccination’ is taken by Navin and Largent to refer to the criminalisation of vaccine refusal. Some take the two terms as synonymous, however; for example, Roland Pierik offers what he takes to be an unqualified defence of what he calls ‘mandatory’ vaccination, which he understands as a ‘non‐negotiable legal obligation’.^4^ This characterization of mandatory vaccination indicates that what Pierik actually means to defend is closer to what Navin and Largent would call ‘compulsory’ vaccination than to mandatory vaccination.

To avoid any confusion and ambiguity, in this article I will adopt and adapt the useful terminology proposed by Navin and Largent: I will argue that vaccination should be compulsory in the sense that it should be *illegal* not to vaccinate oneself or one's children against certain infectious diseases (whether it should count as criminal depends on how one frames the distinction between ‘criminal’ and ‘illegal’, which requires a discussion of legal theory for which I do not have space and competence; I am happy to leave this question open).

Importantly, I am not claiming that my argument applies to all vaccines or even to all the vaccines mentioned at the beginning. Here, I take my argument to certainly apply to a vaccine that protects against a still too common and dangerous infectious diseases like measles, for which the threshold of herd immunity is quite high (as said above, 95%). Whether and to what extent it applies to other vaccines is a question that I am happy to leave open, but for reasons that will become clear below, the lower the threshold for herd immunity is, the weaker my argument becomes.

Before offering my argument, I want to suggest that some of the arguments that might look more intuitive and that have sometimes been used in favour of compulsory vaccination might need to be supplemented by further arguments in order to yield the strongest case possible for compulsory vaccination (Section 2). After that, I will provide my own argument for compulsory vaccination on the basis of the analogy between vaccine refusal and tax evasion (Section 3). Finally, I will address 10 possible objections to my argument (Section 4).

## On Risk‐Based Arguments for Compulsory Vaccination

According to some views, vaccination should be compulsory because (a) non‐vaccination puts people at risk of infection or even death from infectious diseases, and (b) states have a duty to protect citizens’ health.^5^ In fact, everyone is potentially vulnerable whether they are vaccinated or not, given that no vaccine is 100% effective. Thus, there are harm‐to‐others considerations in favour of compulsory vaccination. According to Jessica Flanigan's effective analogy, the state should prohibit non‐vaccination in the same way as it prohibits someone from randomly firing a gun in public space, since the two types of conduct equally put the health or even the life of vulnerable people at risk.^6^ Also relying on considerations of harm prevention, Roland Pierik has argued that the state has a duty ‘to guard the common good of herd immunity’ in order to protect vulnerable people^7^ and that this duty justifies compulsory (i.e. what he calls ‘mandatory’) vaccination policies.

However, with regard to Pierik's argument, we should note that if the aim of vaccination policies is to guard herd immunity, compulsory vaccination is not always necessary. All that is required of states is that, according to a principle of least restrictive alternative that both Flanigan^8^ and Pierik^9^ endorse, they enact the least coercive policy that can guarantee that a sufficiently large portion of the population be vaccinated. And this type of policy need not be compulsory vaccination. For instance, in the United Kingdom, the herd‐immunity threshold of 95% coverage for the MMR vaccine was reached in 2017 without any coercive policy in place. This does not mean that Pierik's argument is not a good argument for compulsory vaccination; quite the opposite. But it does mean that his argument does not work in all circumstances.

Similar considerations can be made about Flanigan's harm‐based argument. Even assuming, with Flanigan, that considerations about risk of harm justify compulsory vaccination, Flanigan's gunfire analogy seems to work well only in cases where vaccination rates are low and therefore the risk of contagion is high: it is only in such cases that being non‐vaccinated is roughly equivalent to firing a gun in a public space. Where herd immunity exists, being non‐vaccinated is more like firing a gun among people wearing good bulletproof jackets or firing a gun in a place where it is very unlikely that vulnerable people will be within shooting distance. The risk is still there, but as the percentage of vaccinated individuals increases, the risk becomes smaller and the gunfire analogy in support of compulsory vaccination becomes weaker. Once again, the validity and the strength of the argument depends on circumstances. According to Angus Dawson, ‘it is hard to see how harm to others considerations can be used to justify an obligation to vaccinate where herd protection does already exist precisely because there will, on the whole, be no additional risk of harm to third parties created by my individual decision not to be vaccinated where herd protection already exists’.^10^ Dawson might be too quick here: some risk of harm might still exist, even if very small (e.g. someone who is infected might enter the community and be in proximity of someone who is not vaccinated). But at the very least, it seems that we can endorse an attenuated version of Dawson's claim that ‘anyone wishing to argue in favour of an obligation to vaccinate where herd protection does exist needs more than [the harm to others principle] to succeed’^11^: we can say that the magnitude of the risk determines the strength of the ‘harm to others’ argument for compulsory vaccination and that in certain circumstances such arguments can become quite weak. At some point, considerations about individual and parental liberty would outweigh considerations about risks of harm.

Nor is appeal to *collective* harm enough to ground a harm‐based argument for compulsory vaccination. At some point, Flanigan seems to be aware that the risk of harm individually imposed by any non‐vaccinated person might not be a strong enough consideration in support of compulsory vaccination, as I have just suggested. To address this objection, she writes that ‘[j]ust because the effect [of non‐vaccination] on herd immunity is small, or imperceptible, does not mean that it is not harmful. Actions that have very small effects, like overfishing or imposing small traffic delays on others, can be extremely harmful in aggregate’.^12^ While the statement about aggregate harm is certainly true, by claiming that the effect of individual non‐vaccination is harmful even if it is imperceptible Flanigan is problematically conflating the notions of individual and collective harm – that is, she is artificially inflating the strength of an argument based on individually imposed risk of harm with considerations that pertain to collective harm. To be sure, it might well be wrong to contribute to a collective harm, but when it is, it is not because the individual contribution is itself harmful. Consider Derek Parfit's famous ‘harmless torturer’ case,^13^ for example. In that scenario, each torturer pressing a button does not make a difference to the pain each victim experiences; it is only the total effect of a significant large number of torturers pressing a button that causes the victims to feel pain. If we want to argue that those who press the button are doing something wrong, or even something that should be legally punished, we need some reason that is independent of considerations of individual harm – as the label ‘*Harmless* Torturers’ that Parfit uses indeed suggests.

One might think that failure to vaccinate violates a ‘clean‐hand principle’, that is, a principle according to which it is not permissible to take part in collective harm.^14^ But this argument seems to be subject to a similar objection: even assuming that the clean‐hand principle is a good *moral* principle, why should we think that it is also a legally *enforceable* principle? If enforceability is only justified by harm prevention, then the enforcement of the principle is not justified, for all the same reasons I have presented above.^15^

Thus, all in all, harm‐to‐others considerations do justify compulsory vaccination when herd immunity does not exist, but they are not sufficient to provide an *unqualified* defense of compulsory vaccination (which is what the title of Pierik's article suggests). Herd immunity minimizes the risks of harm entailed by non‐vaccination, and when the risk is sufficiently small, the violation of individual or parental freedom or of other individual rights like bodily integrity might not be all considered justified, if these individual rights are taken to be sufficiently serious ones. Thus, when herd immunity exists, an additional type of justification is needed. Also, regardless of whether harm‐to‐others considerations provide a sufficient defence of compulsory vaccination, my aim here is to provide an alternative justification that is not meant to necessarily replace the first one. Sometimes there are different kinds of justification for the same conclusion. I propose that an analogy with taxation can provide one such justification.

## On What Grounds Could Vaccine Refusal Be Made Illegal?

Harm or risk of harm to others is not the only acceptable ground for state interventions in contemporary liberal societies, although it might well be the most fundamental. John Stuart Mill famously wrote that the state can legitimately interfere with individual liberty *only* in order to prevent harm to others,^16^ including when harm can be caused by inaction^17^ (as is the case with non‐vaccination, for instance). However, a less frequently cited claim by Mill is the one according to which each person should bear ‘his share (to be fixed on some equitable principle) of the labours and sacrifices incurred for defending the society or its members from injury and molestation’.^18^ Thus, even according to Mill, the ‘harm principle’ might well be the only principle that can legitimately regulate state intervention, but when the harm in question is collective, it seems that it needs to be supplemented by an ‘equitable principle’ in order to determine one's fair share of labours and sacrifices needed to prevent that harm.

Taxation is a clear example of this division of labours and sacrifices. Each individual is required by the state to pay their fair share of taxes, but not necessarily because failing to do so would harm others: unless I am a millionaire, whether I do or I do not pay my fair share of taxes is unlikely to make any substantial difference to whether the state can preserve certain social and public goods. Instead, the reason is simply that it is *fair* that everyone makes her contribution to important social and public goods, quite independently of the actual individual impact.

More precisely, there are three reasons that, taken together, make paying taxes a moral duty for an individual in spite of its negligible impact, at least with regard to taxes used to preserve important public or socially valuable goods (when I talk of taxation from now on, I will refer to taxation for important public or socially valuable goods). First, paying taxes often entails a relatively small cost to individuals, at least when taxation is proportionate; the small cost involved excludes the possibility that paying one's fair share of taxes is supererogatory. Arguably, this is not always the case, and I am happy to leave the question open as to when and why large‐cost taxation would be justified. Here, I am merely referring to taxation involving small individual costs, that is, taxation which would still allow individuals to have enough money to satisfy their basic needs as well as some non‐basic needs and desires (saving money, going on holidays, and so on). It seems that in most cases this is the kind of taxation in place and that this is the type of taxation that most people would agree is ethically justified (though heavier taxation might be justified as well). Second, collectively paying taxes would prevent harm to the community or would benefit the community at a small cost to any individual, so in an important sense there is a collective responsibility to generate enough tax revenue. Third, fairness requires that the burdens of a collective responsibility are fairly distributed among the individual members of the morally responsible collective.

But there are also reasons that justify a *legal*, and not only a moral, duty to pay taxes: when the public and the collective goods at stake are important for the functioning and upkeep of society, it is reasonable to *legally* require individuals of a community to fulfil their moral obligations. Paying taxes in order to fund these goods is not only a moral duty, but it should also be – as in fact it typically is – a legal duty. With the exception of certain libertarian circles, the idea that it is reasonable to legally require people to pay taxes – at least those needed to fund ‘presumptively beneficial’ goods^19^ – is relatively widespread and considered uncontroversial.

Compulsory taxation is consistent with the promotion of *both* (a) *expected utility*, in that it allows the realisation of important social and public goods essential to the survival and wellbeing of society, and 2) *fairness*, in that it ensures that each individual makes her fair contribution to such goods. Utility and fairness are closely related in the sense that a fair system of distribution of burdens would allow to solve the so‐called ‘problem of assurance’, that is, the collective action problem that arises from the fact that people would feel less motivated to make their contribution to a certain good that is beneficial for the collective if they do not have enough reassurance that other people would contribute as well.^20^ To solve collective action problems of this type, it is often necessary to use some coercion by an authority. The justification for state coercion based on the problem of assurance is the stronger, the more important for the upkeep of society the goods promoted by taxation are. For trivial goods such as, for instance, firework shows, it is more difficult to justify coercive taxation: the mere assurance that other people will contribute to the good because coerced does not seem to be enough.

Now, herd immunity also belongs to the category of goods essential for the preservation and the upkeep of society and that a community has a duty to preserve. Herd immunity is a public good,^21^ in the sense that it is non‐excludable and non‐rivalrous in consumption: it is impossible, or it is extremely difficult, to exclude people from benefiting from the good, and any individual benefiting from the good in question does not diminish the extent to which other people would benefit as well. Because vaccines are typically not 100% effective, even a vaccinated individual might benefit from herd immunity to gain protection from infectious diseases. Moreover, many benefits of herd immunity are indirect and are enjoyed also by those who are effectively immunized: herd immunity promotes other important public goods that are essential to the upkeep and survival of society, such as a reduced burden on a public health system and even political stability and national security, which in extreme cases could be compromised by infectious disease outbreaks.^22^ For example, a pandemic of simple seasonal influenza could cost a country like the United States a loss in gross domestic product of $ 45.3 billion.^23^

The non‐excludability of a good like herd immunity gives rise to a free‐riding problem: it is in any individual's self‐interest to benefit from herd immunity without bearing the burdens of vaccination (however small these are), but too many people acting in their own self‐interest would compromise the good in question. And even when free‐riders do not compromise the good because there are few of them, they clearly seem to be violating a requirement of fairness, to the extent that we think free‐riding is unfair. Besides, even if someone did not *intend* to free‐ride or did not in fact free‐ride because there is nothing to free‐ride on where herd immunity does not exist, the non‐vaccinated would still be violating a different requirement of fairness, namely a requirement to make one's fair contribution the fulfilment of a collective obligation, in this case the collective obligation to preserve an important public good like herd immunity.^24^ What ‘collective obligation’ precisely means is in itself a complex philosophical issue, which I do not have space to explore here. Elsewhere, I have discussed how the notion of collective responsibility applies to the case of vaccination and herd immunity.^25^ With reference to that previous discussion, by ‘collective obligation’ I mean here that there is an obligation that can only be fulfilled through the *aggregate* actions of (at least some of) the members of a certain collective.

Now, the central claim of this article is the following: *vaccine refusal is analogous to tax evasion* in those aspects that justify compulsory policies. In both cases an individual fails to make her fair contribution to socially valuable goods, even if only a negligible contribution.

The duty to vaccinate is a moral duty for the same reasons I have provided above about taxation.

First, vaccination normally entails a *small cost* to individuals – actually, a much smaller cost than taxation – which once again rules out the possibility that getting vaccinated is supererogatory, and therefore it prevents a reason against a moral duty to be vaccinated. The only case in which vaccination should not be seen as a moral duty is when it actually would be supererogatory, for example in the case of vulnerable individuals for whom vaccination is medically contraindicated. Importantly, we need to distinguish between a subjective and an objective cost of vaccination: the former refers to the psychological consequences of vaccination, in particular for someone who has a strong moral or religious opposition to it; the latter is the cost in terms of vaccine injuries. I submit that the morally relevant cost in determining whether vaccination is supererogatory is the objective one: if subjective costs were factored in, then basically anything could be considered over‐demanding at least for someone, with the undesirable consequence that one's personal moral or religious views could exempt anyone from any moral obligation. Granted, moral or religious opposition might be relevant to the issue of conscientious objection to compulsory vaccination, which I will address towards the end of this article, but it is important to point out that if conscientious objection is legitimate, it is because of a principle of freedom of conscience, not because of the demandingness of acting against one's own conscience. Again, the analogy with taxation can help us clarify the point: the fact that some people might be strongly morally opposed to certain taxes (e.g. those used to fund abortion services) is normally not taken to be a good reason against a moral duty to pay taxes, although it might be a good reason for allowing conscientious objection to compulsory taxation,^26^ as we shall see below. I should also clarify that the (objective) cost of vaccination is small in the sense that the probability of serious (that is, life threatening or causing permanent damage) side effects is extremely small; according to the WHO, ‘so few deaths can plausibly be attributed to vaccines that it is hard to assess the risk statistically’, and ‘serious adverse events occur rarely (on the order of one per thousands to one per millions of doses)’.^27^ Admittedly, however, the cost will not be small for the few unlucky ones who will experience such side effects. So the previous claim should be qualified as follows: vaccination entails a small cost *ex ante* (because of the extremely low probability of serious injuries), but in extremely rare cases it might entail a high cost *ex post facto*. However, it is the *ex ante* cost that matters for the purpose of determining the level of demandingness when the probability of a large *ex post facto* cost is very low. After all, this is the same standard we normally adopt to establish the low level of demandingness of other normally accepted ethical and legal requirements. Wearing seat belts and buckling up one's children, for instance, entails a very small *ex ante* cost but can in very rare cases have significant *ex post facto costs* in the form of severe seat‐belt injuries, which can sometimes be more serious than the car accident injury they prevent.^28^ Sometimes we do consider policies with small individual *ex ante* costs and very large individual *post facto* costs impermissible, though. I will address this point in the section dedicated to meeting objections below.

The second reason why vaccination is a moral duty is that individually it can prevent infecting others and collectively would allow achieving herd immunity; harm prevention and benefit to others represent moral reasons in favour of adopting a certain behaviour, and therefore, in this case, they ground a *collective* moral responsibility to realise herd immunity.

Third, and closely related to the second reason, fairness requires that the burdens of the collective responsibility to realise herd immunity be fairly shared among the individual members of the morally responsible collective.^29^ As said above, the fairness requirement could take the form either of a requirement not to free‐ride on herd immunity and/or a requirement to make one's fair contribution to the fulfilment of a collective responsibility.

Importantly, the moral duty to be vaccinated should become a legal duty for the same reason why paying taxes is a legal duty. As I have said above, the public good of herd immunity is essential to the preservation, upkeep, and wellbeing of a community. And compulsory vaccination would promote both *expected utility*, by realising an important public good, and *fairness*, by ensuring that each individual makes her fair contribution to herd immunity. Also in the case of vaccination, a legal obligation to vaccinate would go a long way towards solving the problem of assurance.^30^ It is important that a policy based on fairness is effective at addressing the problem of assurance because the very notion of fairness implies a comparison with other people's contributions. If too many people do not make their own contribution, my duty of fairness is significantly weakened. Thus, in an important sense an argument for compulsory vaccination based on fairness depends on circumstances, as was the case with harm‐based arguments: my duty of fairness only exists to the extent that I can be assured that other people can contribute as well. Compulsory vaccination, if properly implemented, can provide such assurance.

Interestingly, Mark Navin did consider the unfairness of free‐riding on herd immunity as a reason for a parental *moral* duty to vaccinate one's children.^31^ As he puts it: ‘members of a community that possesses herd immunity have a moral reason of fairness to contribute to their community's herd immunity’.^32^ However, like other authors, he also takes state coercion to be limited by a principle of least restrictive alternative; according to Navin, ‘the state has the authority to coerce vaccination, though there are good reasons for it to use as little coercion as is necessary to achieve the goal of herd immunity’.^33^ Thus, although fairness is for Navin a very relevant consideration in the justification of moral duties, it is outweighed by the principle of least restrictive alternative when it comes to justifying the legal enforcement of such duties. However, if my analogy with taxation works, it is not clear why the principle of least restrictive alternative should outweigh considerations of fairness in the case of vaccination, when it does not do so in the case of taxation. I will explore this point in more detail when addressing objection 4 in Section 4.

Likewise, the fact that a certain community would or would not achieve herd immunity regardless of whether any one individual or a small proportion of individuals vaccinate their children does not seem to provide a sufficient reason for exempting certain individuals from the vaccination requirements. A sufficiently low rate of tax evasion does not make a significant difference to the economy of a country. Still, the insignificance of any individual contribution or of a small group's contribution to one's society through taxes does not provide a strong enough reason for exempting a minority of individuals from taxation requirements. The same should be said about vaccination. In both cases, it seems that fairness is a fundamental value that we want to uphold, quite independently of consequentialist considerations.

## Meeting Ten Objections

I will now address 10 likely objections against my argument. The first seven objections target the analogy between taxation and vaccination (that is, they are not necessarily arguments against compulsory vaccination, but only against the analogy as a good reason for compulsory vaccination): objections 1–5 point to relevant disanalogies, while objection 6 and 7 point to the fact that the analogy, even if valid, does not necessarily imply the same legal treatment. The last three are objections to compulsory vaccination in itself. For the objections that target compulsory vaccination in itself, sometimes the taxation analogy helps addressing them. For the objections that target the analogy with taxation, I will suggest that they probably do not undermine the case for compulsory vaccination based on the analogy, although some might at least in certain circumstances significantly weaken it. My modest claim is simply that the reasons for compulsory vaccination based on the taxation analogy seem to outweigh the reasons against based on disanalogies. The objections are the following.

1. *Adult vs. child obligations*. Most vaccination decisions concern young children, who are not competent moral agents. This makes it problematic to argue that they are subject to a moral obligation of fairness or to a legal obligation to make their fair contribution to a public good. After all, with reference to the taxation analogy, children are normally not morally or legally required to pay taxes.

There can be a paternalistic justification for imposing vaccination on children – namely, a form of impure paternalism where the subject being coerced (e.g. parents) is not the same as the subject who benefits from the policy (e.g. children)^34^ – but where herd immunity already confers children indirect protection, can vaccination be imposed on children regardless of whether they stand to benefit from it? Now, certain vaccinations are indeed recommended for children who arguably are old enough to be considered competent moral agents and who can be expected to make their fair contribution. For example, vaccines against meningococcal groups A, C, W, and Y disease are recommended for 12‐year‐old children, who arguably do count as moral agents and are subject to moral and legal obligations; the same applies to the seasonal flu vaccine which adults can decide to get every year. But what about younger children and infants, who are the ideal target of the MMR vaccine? I have three responses to this objection.

First, with regard to moral obligations in the case of very young children, the fairness‐based obligation in such cases is not that of being vaccinated that would fall on young children, but that of vaccinating one's children that falls on parents. After all, parents make many decisions on behalf of their children, and there is no reason to think that *moral* decisions should not be among these, especially when the moral decision entails a very small cost to children (and it is also worth mentioning that paying taxes might negatively impact one's child as well by taking away resources that could have been spent on the child). Now, if the cost imposed through proxy decision‐making were large, then the fact that the cost is on a third party (the child) rather than on those who make the moral decision on their behalf (the parents) would create problems for the legitimacy of proxy moral decision‐making; to many people, it is problematic to impose significant risks on those who cannot consent to abiding by certain moral requirements that entail such risks. In any case, since, as I have argued above, what matters for the purpose of determining the demandingness of a course of action is the *ex ante* cost, and the *ex ante* cost in the case of vaccination is very small, proxy moral decision‐making does not seem ethically problematic in this case.

Second, the choice not to vaccinate one's child is a proxy decision that exposes the child to risks (of infectious diseases), so it seems that there is no way of avoiding some risks through proxy decision‐making – and actually vaccination is the choice that exposes the child to lower risks.

Third, with regard to legal obligations, the reason why children are not legally required to make their contribution to society through taxation is that it is unreasonable to expect them to become contributors to society's upkeep. However, in the case of vaccination, they can easily make their contribution by being vaccinated without experiencing any unreasonable cost, so there is no reason to think that they may not be subject to the legal obligation to be vaccinated. It is true that while in the case of taxation the subject of coercion is also the one who bears the cost, in the case of compulsory childhood vaccination the subject of coercion (normally the parents) is not the one who enjoys the most benefits. This fact clearly represents a disanalogy between the two cases. But it does not seem that such disanalogy is fatal to an argument for compulsory vaccination based on the analogy between taxation and vaccination. It is often the case that parents are coerced into acting in their children's best interest, with the only difference that in most cases, if they fail to do so (e.g. they fail to provide adequate medical care or proper nutrition), interventions are ordered and enforced by authorities (think, for example, of forced blood transfusions for children of Jehovah's Witnesses). The vaccination policy I am arguing for here is even milder than such policies, since I am not talking of forced vaccination. Therefore, it seems, it is even more strongly justified.

It is important to point out that I am not reintroducing a harm‐prevention argument in support of compulsory vaccination. The point I am making is simply meant to defuse the relevance of a disanalogy between compulsory taxation and compulsory vaccination: while I acknowledge that the fact that, in the vaccination case but not in the taxation case, those who are coerced are not necessarily the same as the ones that bear the main consequences of coercion, I submit that such disanalogy does not undermine the case for compulsory vaccination based on that analogy. The reason is that the disanalogy concerns a factor that is normally not taken to undermine the justifiability of compulsory health policies, if the policy is in a child's best interest. Thus, the disanalogy exists but it is irrelevant. I am not making any claim here about whether being in a child's best interest is enough to justify compulsory vaccination. I have expressed my doubts about this in Section 2. In any case, my argument is not based on it. Here, I am only using the notion of ‘best interest’ to make a disanalogy between compulsory vaccination and compulsory taxation less salient.

- ‘*Making a difference*’. One might observe that any individual not paying their fair share of taxes would still make a slight difference to government revenue and therefore the more money a government receives, the better. In other words, it is still preferable that one more rather than one less individual pays their, however small, fair share of taxes. On the other hand, herd immunity represents a precise threshold above which any additional vaccination would not make any difference whatsoever to whether the public good obtains. Both observations are correct, but they refer to different senses of ‘making a difference’. Once these two senses are clarified, I think it is possible to show that taxation and vaccination make or do not make a difference in the same way.

The first sense does not consider thresholds: if it is true that any individual contribution to government revenue through taxes does make some difference, however small it is, to government revenue, so that the more the better, then it is also true that any individual vaccination does make a small difference to minimization of risk of infection whether or not there is herd immunity. For instance, someone might still find herself in proximity of an infected individual who contracted an infection elsewhere and brought it into the community, or vaccination rates might drop below herd immunity at any time, and any vaccination might contribute to preventing risks of contagion. The risk can be extremely low – as is the risk that a tax evader would make a significant difference – but is never zero.

The second sense does take thresholds into account, and again it applies equally to both cases: if it is true that any additional vaccination above the herd‐immunity threshold does not make a difference to whether the public good in question obtains, then it is possible to identify an equivalent of herd immunity for many other public goods funded through taxes (e.g. national defense). This would be the minimum sum of revenue necessary to guarantee that the good obtains or that it is up to a certain standard, above which any additional revenue would not make a difference to whether the good obtains or whether it obtains to that standard.

Therefore, in terms of ‘making a difference’, it seems that vaccination and paying taxes are on equal footing and that ‘making a difference’ is not a criterion for treating them differently, either morally or legally. Furthermore, even assuming that there is a sense of ‘making a difference’ whereby taxation does and vaccination does not make a difference, it is worth remembering that my argument is based primarily on considerations of fairness. While ‘making a difference’ matters a lot in the case of harm‐based arguments (which I have discussed at the beginning), it seems not to matter much, or even not to matter at all, in the case of arguments based on fairness.

- * Side effects and responsibility for them*. A third objection to compulsory vaccination is that there is a small chance of significant negative side effects in the case of vaccination, but not in the case of taxation. I acknowledge that this aspect does represent a disanalogy with the case of taxation, or at least with the case of taxation that does not itself harm significantly tax payers or their family members. Admittedly, taxation can be too high and therefore harmful to some people, but in most cases it is not. Even when taxation is, say, 50% of a worker's income, it is unlikely to significantly harm tax payers: they would normally still be able to have a good life and to live well above the threshold of ‘having enough to survive’ (but even if a 50% taxation is harmful, it is often considered acceptable, which if anything supports the taxation analogy as a kind of argument for compulsory vaccination in spite of the risks of vaccines). The question is whether the disanalogy is relevant enough to undermine the case for compulsory vaccination.

There are two issues at stake here: one is the imposition of risks of harm through vaccination, and the other is attribution of responsibility (and legal liability) for such harms, when they materialize.

Let us start from the first issue. The risk of harming the child exists both in the case where the child is vaccinated and in the case where the child is not vaccinated. Measles is fatal in 1 in 100 cases in poor regions of the world and in 1 in 5,000 cases in high‐income regions, but it can also lead to serious complications such as pneumonia and encephalitis (inflammation of the brain), which consequence permanent damages.^35^ In contrast, the MMR vaccine is extremely safe: anaphylactic reactions, the most serious side effects, happen in less than one in a million vaccinated individuals.^36^

These data raise a question, related to the second aforementioned issue, namely attribution of responsibility: even if a very small risk of side effects of vaccines exists, what is the alternative to individuals running these risks and to the state being liable for these side effects? The alternative is that someone else, namely the parents who refuse vaccination, would arguably be morally responsible for the possible bad consequences of non‐vaccination either for their children or for the vulnerable members of a community who cannot be vaccinated and whom their children infect.

One can observe that there might be a moral difference between generating risk of harm by omission (i.e. by failing to vaccinate) and generating risk of harm by action (i.e. by vaccinating); in fact, many parents who are hesitant about vaccinating their children claim that they would feel more responsible if something happened to their children as a consequence of vaccination than as a consequence of non‐vaccination.^37^ Even assuming that this attitude is not irrational and the act‐omission distinction is an at least reasonable moral position, it seems plausible to assume that, when we move from the perspective of individual action to that of government action, a policy should aim primarily at benefiting the community and individuals, provided this can be done fairly. Governments are not moral agents in the same way as individuals are: they exist mainly to fulfil certain functions, such as preserving public health. Public policies are of course subject to deontological constraints, but because governments exist primarily to fulfil certain functions, the way such constraints apply to government action is not necessarily the same as the way they apply to individual action. In the case of public health policies, it seems the priority of the state should be to guarantee a good level of public health through fair legislations, assuming this does not violate any *significant* deontological constraint. Since children will run some risk either way, i.e. with or without vaccination, and since unvaccinated children where vaccination rates are low pose a risk to vulnerable individuals, it seems that the priority should be to minimize the risk, not to shift moral responsibility and legal liability away from the state. Even if there often are deontological constraints in terms of act‐omission distinction on state action, these do not seem to be too *significant* in the case of vaccination policies, regardless of how significant they are taken to be in the case of individual action. This is because, even if the act‐omission distinction has some moral relevance in some or even most contexts, it seems plausible to assume that the easier and less costly the act in question is, the less morally significant its difference with ‘doing nothing’ is. This claim would require some arguments for which I do not have space, but elsewhere^38^ I have argued that the moral relevance of the act‐omission distinction can plausibly be explained by the fact that acting is typically more difficult and more demanding than doing nothing, and therefore it seems intuitive to many people that any harm resulting from action, requiring some significant conscious effort by an agent, is morally worse than any harm resulting from inaction. People might still hold on the act‐omission distinction when making individual decisions, but government action needs to abstract from individual takes on the act‐omission distinction. Since vaccination is an easy and costless option, it seems the moral relevance of the act‐omission distinction fades away when we compare the harms of non‐vaccination with the (unlikely) harms of vaccination. Thus, the state cannot be considered morally responsible for imposing such risks if the imposition of risks is morally justified. Vaccine injuries will probably occur if millions of children are vaccinated, and in such cases victims would be morally entitled to some form of compensation, for example through vaccine injury compensation funds^39^ which are in use in many countries, such as Scandinavia countries, the United States, and the United Kingdom.^40^ But that they are owed compensation does not mean that the imposition of the risk was ethically impermissible and that the state is morally responsible for it, *all things considered*. Simply, sometimes bad things happen without anyone being morally responsible for them.

- *Side effects: a reappraisal*. One might reply here – and this is a fourth objection – that where herd immunity exists, the risk that a non‐vaccinated child becomes infected and experiences some particularly negative consequences of the infectious disease might be smaller than the risk – however small this is – that the child experiences some side effects of the vaccines. Fairness does not seem to constitute a strong enough reason to expose certain people to additional risks without any prospect of benefit.^41^ I have three answers to this objection.

First, it is dubious that vaccination would not benefit an individual where herd immunity exists: the individual would gain individual immunity, which would protect her if she ever finds herself within a community where herd immunity does not exist (either because the vaccination coverage in her community drops or because she moves to a different community), or if she is exposed to someone who is infected. Arguably, I can be said to benefit from an intervention even if the benefit takes the form of protection from future risks, rather than from risks at present. In the case of vaccination, in particular, this type of benefit is even more apparent, considering that if and when vaccination coverage drops below the herd‐immunity threshold, it might be too late for the unvaccinated individual to get the vaccine and gain protection. Actually, we often realise that vaccination rates have fallen below the herd‐immunity threshold precisely because cases of infections increase. Thus, it is dubious that the risk of vaccines would constitute an all‐things‐considered burden to the individual. Thus, it is plausible to suppose that there are (impurely) paternalistic reasons to coerce vaccination on children even where there is herd immunity, or at least that such reasons can be used to prevent the objection I am addressing here.

My second reply is that, even assuming for the sake of argument that the risks outweigh the benefits, how are we supposed to pick out those privileged people who are allowed to free‐ride on herd immunity? Even in contexts where herd immunity already exists, there is no guarantee that it will last over time, since vaccination rates might drop at any time. Compulsory vaccination can prevent such drop, but why should those who happen to live in a community that at a given time has herd immunity have the privilege of not contributing their fair share towards the preservation of such an important good? Leaving aside for the moment a seemingly plausible answer, namely a lottery solution (I will discuss it below), these questions seem to suggest that fairness in distribution of risks is a relevant consideration that might justify at least a very slight increase of risks.

Which brings me to my third reply: it is true that fairness comes at the cost of unnecessarily multiplying some small risks, but what is the actual cost of fairness in terms of additional risks? The word ‘additional’ is important here: the morally relevant risk is not the one imposed on the whole population, but that imposed on those who would not get vaccinated if the principle enforced was not one of fairness but merely one of harm prevention. In other words, we have to assume as baseline the risks of vaccines that would anyway be involved by the mere realization of herd immunity through less coercive means. Fairness imposes an extremely small risk on a very small additional population, which is likely to make the additional risk insignificant. Take measles. Herd immunity requires 95% vaccine coverage: within such a high percentage of vaccinated individuals, there might be a few cases of adverse vaccine side effects that are normally not taken to be good enough reasons against policies aimed at realizing herd immunity (whether coercive or not). With compulsory vaccination, the vaccination coverage rate would probably be higher, though not 100%: there will always be people who can't be vaccinated for medical or age reasons, and presumably – as happens with tax evasion – there will always be a fraction of the population that gets away with escaping vaccination requirements. Considering how small the risk of serious side effects is, it is very likely that the difference in risks between 95% coverage and *x* coverage with 95% < *x *< 100% is negligible and likely outweighed by considerations of fairness, absent any fair criterion for exemptions. After all, we are talking of an extremely small risk (of the magnitude of 1 in 1 or 2 million) on a very small additional population (presumably 2–3%). Admittedly, though, the lower the threshold of herd immunity for a certain infectious disease is, the weaker my response to the objection becomes. Thus, the strongest case I can provide for compulsory vaccination only applies to certain vaccinations but not to others, which is why I said at the beginning that my argument is meant to apply to a vaccine like the MMR vaccine, but that I am happy to leave open the question whether it also applies to other vaccines. For some vaccines, the disanalogy with taxation in terms of risk imposition might become significant.

- *Bodily integrity*. A fifth objection to the analogy with taxation is that vaccination violates a right to bodily integrity, and therefore there are reasons not to compel people to be vaccinated, while there is no comparably strong right that taxation would violate. This certainly represents another disanalogy with the case of taxation.

The right to bodily integrity is quite peculiar to compulsory vaccination and is far less controversial than any right taxation might be taken to violate, such as, according to some libertarians, a right to private property. Admittedly, the right violated in the case of compulsory vaccination seems more stringent than whatever right (if any) is violated in the case of compulsory taxation (unless one is an extreme libertarian). Thus, the strength of this objection rests on how stringent one thinks the right to bodily integrity is. For those who think the right is almost an absolute one, the disanalogy would probably undermine the case for compulsory taxation. However, it is doubtful that a right to bodily integrity is such a preponderant moral consideration, especially when we talk of individuals who cannot give valid consent. We do allow violations of bodily integrity both on non‐competent and on competent individuals when something sufficiently valuable is at stake: necessary blood transfusions or other beneficial medical treatments on non‐competent children are normally ordered by courts, and rightly so; vaccination or other prophylactic medical treatments for certain categories of people, e.g. health workers or travellers, are normally mandated, and rightly so. In both cases, such requirements do violate bodily integrity to protect individuals’ health but often also to protect other people (such as in the case of vaccinations for health workers or travellers).

So, for consistency's sake, we should say that compulsory vaccination is justified in spite of the violation of bodily integrity at least where herd immunity does not exist, because it protects the vaccinated individual and it protects others. But we said above that vaccination is in a child's best interest even when herd immunity exists. Violating someone's bodily integrity in a way that benefits them (regardless of whether the intervention is intended to benefit them or is undertaken in the name of some other value, such as preserving public goods or fairness) seems acceptable at least when the individual in question is not deemed competent to make a decision as to whether or not to accept the intervention. Again, it is worth repeating that while a child's best interest is not the principle that supports the main argument of this article – the principle is fairness – it can be used to defuse the salience of disanalogies between compulsory vaccination and compulsory taxation, once these have been acknowledged.

Also, fulfilling a fairness requirement might well be an additional reason that outweighs the moral value of bodily integrity, especially when the fairness requirement helps solve the problem of assurance and therefore makes herd immunity more likely: even conceding, for the sake of argument (and this is quite a big concession), that the intrinsic value of fairness cannot outweigh the value of bodily integrity, its instrumental value probably can.

- *Conscientious objection and conscription*. In the case of military conscription, conscientious objection to contributing to national defence is, or was – when conscription was common in many countries – often granted. For the sake of argument, I will assume that conscientious objection to the military service is justified. Thus, while the analogy with tax evasion seems to rule out conscientious objection to vaccination legal requirements, the analogy with conscription suggests the opposite. So, what are the reasons to prefer the former to the latter analogy?

Actually, one could push the objection even further. Jason Brennan^42^ has argued, that there is no reason why, in liberal societies at least, conscientious objection to fighting in war should be accommodated while ‘conscientious tax objection’ should not: the two stand or fall together. In his view, the four reasons that, at least in liberal states, justify the former equally apply to the case of taxation. Such reasons are the promotion of justice and rights, protection of state stability, respect for ideological minorities, and state accountability to reasonable people in spite of disagreements. If Brennan's argument works (which I will not discuss here), no matter which analogy one chooses (either taxation or the military service), it would still support the case for conscientious objection to compulsory vaccination based on the same four reasons.

However, it is worth noting that protection of individual conscience in the military case should not, and in fact it typically does not, supersede the fairness requirement; it merely redirects it towards available alternative forms of contribution, as Brennan himself acknowledges.^43^ In fact, being granted conscientious objection to military conscription typically comes with a requirement – based on fairness – to provide some alternative contribution to society that is roughly equivalent, also in terms of personal sacrifice, to the contribution one makes through the military service. Whether or not Brennan's point implies that this should apply to taxation as well, some have suggested that the same requirement should be applied to conscientious objection to vaccination^44^ But if so, then it means that *in an important sense* vaccination remains compulsory even if we allow conscience exemptions: people are compelled to make an alternative but roughly equivalent contribution to some roughly equivalent public good for the sake of fairness precisely because they are expected to make their fair contribution to that public good in the first place. The problem is whether it is possible to make a contribution that is roughly equivalent to vaccination, especially in situations in which herd immunity is threatened. In the case of the military, there are many other socially useful things that a state needs people to do in order to guarantee the upkeep of society, and without which national security itself would be purposeless: even in times of war, we only need at most a small fraction of a population to be conscripted, because other vital public goods and social functions need to be maintained. Thus, it makes sense to say that the alternative contribution required of a conscientious objector to the military service is roughly equivalent to the contribution to national defence made through military service. But with regard to vaccination, the public good in question is so specific and requires that specific contributions (vaccination) from so many people that it is hard to imagine what would count as roughly equivalent. Thus, conscientious objection to compulsory vaccination does not seem a viable option, even if it is in the case of the military and perhaps in the case of taxation. Granted, one equivalent alternative would be to simply confine people who refuse vaccination. But voluntary confinement as a penalty for conscientious objection would hardly be chosen by anyone, and it would not be significantly different from an extreme form of punishment. People would probably prefer most other penalties for non‐compliance with compulsory vaccination policies, such as fines, unless these involved prolonged jail time.

- *Alternative fairness requirements*. One might observe that there are alternative ways of fulfilling a fairness requirement. One example would be a tax to support public immunization programs – call this the ‘immunisation tax’. Consider the case of a different public good like public safety: fairness does not imply that everybody has a duty to serve in the police force; instead, everybody ought to pay taxes such that the police service in a country can be maintained.^45^ Why, then, should we require people to vaccinate, instead of simply requiring them to pay taxes towards the implementation of vaccination policies that would realise herd immunity?

My answer here resembles the one I gave to the previous objection: paying the tax does fulfil one's duty of fairness as I have characterised it here in the case of public security, but not in the case herd immunity. Paying taxes instead of being vaccinated might satisfy some fairness requirement,^46^ but not the requirement to make one's fair contribution to herd immunity, because there is no guarantee that with an immunisation tax there would be enough people willing to be vaccinated. In the case of public security through police, instead, the tax would certainly contribute towards public safety, because it would fund salary for police officers and therefore create an incentive to serve in the police that would ensure that there are enough people in service.

The only way to sustain the present objection would be to argue that being vaccinated or vaccinating one's children, exactly like being a police officer, should be seen as a service for which someone needs to be paid, that people would not get vaccinated or vaccinate their children unless they are paid (as is the case with most police officers), and that we are likely to find a sufficiently high number of individuals who are willing to be vaccinated or to vaccinate their children in return for some payment. This claim, however, raises a separate issue about whether we should pay people to be vaccinated, which is beyond the scope of this article.^47^

An alternative way of implementing a fair policy that guarantees protection through herd immunity is having a fair lottery that would exempt from vaccination requirements the largest number of people, e.g. 2% of the population, that would not compromise herd immunity even if they weren't immunized. At a first glance, this variation of a compulsory vaccination policy seems to be preferable to my alternative because it interprets fairness in a way that allows applying a less restrictive but equally effective policy (as long as the target is herd immunity), thus going further than my proposal in reconciling fairness, expected utility, and the principle of least restrictive alternative. I am happy to concede that this is a preferable option, but only if we accept – as I suppose many people would not do – that an analogous policy should be adopted in the case of taxation. For what I have said at the end of Section 3, individual contributions to public goods through taxation and through vaccination are analogous in terms of ‘making a difference’; hence, there must be a small number of individuals (even if it was just one individual in a country) that could be exempted from taxation requirements without impacting the state's capacity to guarantee certain goods at a certain level. If we are prepared to accept the idea of an exemption lottery for taxation, I am happy to concede that the exemption lottery for vaccination would be a preferable alternative to compulsory vaccination for everyone. I suspect, however, that most people would not accept the antecedent.

- *Back‐firing*. A possible objection to compulsory vaccination is that coercive policies might back‐fire, for example, ‘by alienating parents and increasing wariness of vaccines’.^48^ This objection does not depend on the taxation analogy. It is an objection to compulsory vaccination regardless of the reason in its support. However, as I will show, the taxation analogy can help address it. The idea here is that if parents were coerced into vaccination, they would become suspicious of the medical and health system establishment, e.g. because they would think that if something is really good for a person, there would be no reason to coerce people into doing it. Now, if the back‐firing is referred to parents’ attitudes, then the objection hits the target, but this should not worry us too much. What matters is the back‐firing in terms of reduced vaccination uptake as a consequence of parents’ alienation and increased wariness. With compulsory vaccination *properly implemented*, there would be no room for such back‐firing. There will always be a penalty parents would not be prepared to pay for their choice not to vaccinate. The problem is that of finding out what that is, which admittedly could require some trial policy, and to implement adequate policing. In fact, less than one year after the implementation of compulsory vaccination in Italy, vaccination uptake increased significantly *likely as a consequence of the policy*, with the actual effect of the law likely to be even greater than the estimated one.^49^

In any case, I am here only trying to establish an ethical principle in support of compulsory vaccination; policymakers are best suited to respond to back‐fire objections once ethical principles are established. And it is worth noticing that the taxation analogy can help address this objection: coercive measures can always alienate individuals and erode their motivation to contribute to the public good. For example, the same argument seems to suggest that coercive taxation policies – say, with high penalties for tax evasion – might back‐fire in the same way. But such consideration would probably not be considered valid reason against compulsory taxation anyway. Simply, the penalty would have to be such that people would have to pay taxes, or to vaccinate their children, even if this meant doing this reluctantly.

Other measures, of course, would need to be implemented to persuade parents that vaccination is the right choice regardless of the legal requirement (e.g. information campaigns, assuming these are effective), but these other measures are compatible with compulsion, not necessarily an alternative to it.

- *Least restrictive alternative*. Someone might object that compulsory vaccination is not consistent with a principle of least restrictive alternative (PLRA) in public health.^50^ Measures less coercive than outright compulsion might be equally effective at realising herd immunity. As some have argued, when this is the case, such less restrictive measures ought to be preferred.^51^ Now, the PLRA would be incompatible with compulsory vaccination only if the only worthwhile aim of a vaccination policy were the realisation of herd immunity. But as I have suggested through my taxation analogy, effectiveness or expected utility are not the only values to be promoted. In some cases, we want to promote also fairness, which is valuable in itself but also instrumentally in order to solve the problem of assurance. One such case is taxation. Since vaccination is analogous to paying taxes in that both represent a fair contribution to important social or public goods, fairness matters in the case of vaccination inasmuch as it does in the case of taxation, both intrinsically and instrumentally. And chances are that the standard of fairness I have argued for can only be guaranteed through compulsory vaccination.^52^ Thus, if the desired goal is not only harm minimization, but also fairness in the distribution of certain burdens, correct application of the PLRA does not rule out compulsory vaccination.
- *Postfacto costs*. Again with regard to the costs of vaccination, I have said above that one of the reasons in favor of compulsory vaccination is that, even if vaccination can entail a large *post facto* cost, it is the small *ex ante* cost that matters for the ethical justification of coercive policies. Some might reply that this is not true.^53^ Sometimes policies with large *post facto* costs, such as John Harris’ survival lottery,^54^ are considered impermissible. In the survival lottery, Harris asks us to imagine a case in which two or more individuals need a different organ each to survive, and a certain computer randomly selects a person among a large population that would be killed in order to provide the organs. In this case, even if the *ex ante* cost is small (given the very, very low probability of being selected) and only the *post facto* cost (being killed) is very large, we would not think that a policy based on the survival lottery is permissible. Admittedly, with regard to the size of risks and benefits involved and their distribution, the survival lottery seems similar to the case of compulsory vaccination. Why should we consider the latter permissible but the former impermissible?

My answer is that the *post facto* cost is not necessarily itself the reason, why policies like the survival lottery are impermissible. It depends a lot on other circumstances. In particular, there is a morally relevant disanalogy between the vaccination case and the survival lottery case in terms of ‘double effect’ and of using people as mere means. With the survival lottery, we pick someone randomly who we intentionally harm in order to benefit others: the positive effect is achieved by intentionally using a negative effect as a means. With coercive vaccination, someone will be harmed unintentionally (even unforeseeably) in order to benefit others. The fact that the survival lottery is impermissible but other policies with high *post facto *costs – such as seat‐belt requirements, to use a previous example – are not suggests that it cannot be the *post facto* cost itself that makes a decisive moral difference. The intentional nature of the harm in the survival lottery case, and the fact that in that case harming people is the means to obtain the positive effect, is likely to play a role in explaining why the survival lottery is impermissible. As said above, even if deontological moral constraints apply differently to individuals and to government, there are some *significant* deontological moral constraints to state action.

There might be other or alternative explanations, of course, as the ‘doctrine of double effect’ is notoriously subject to criticism, especially from the consequentialist camp. My point is simply that the large *post facto* cost cannot make the whole moral work in explaining why the survival lottery is impermissible and that appealing to double effect could do at least part of the work. In other words, the magnitude of the *post facto* harm might be necessary to explain why the survival lottery is impermissible, but it does not seem to be sufficient. Thus, it cannot be sufficient to make compulsory vaccination impermissible either. With respect to the ‘intended/foreseen’ distinction and to the ‘mere means’ criterion, compulsory vaccination is similar to seat‐belt requirements (considered acceptable) rather than to the survival's lottery (considered unacceptable). At the same time, however, I am not suggesting that the intended/foreseen distinction and the ‘mere means’ criterion are sufficient to make a policy with a very bad intended outcome impermissible either: a policy similar to the survival's lottery but with small individual *post facto* costs could be permissible even if it involved intentionally harming someone, but only if the good at stake is important enough and/or the individual cost is small enough to outweigh the moral relevance of intentionally harming someone. And when the harm to someone is not even intentional, as in the case of vaccination, this last claim seems even stronger.

## Conclusions

I have argued that a case for compulsory vaccination cannot rest solely on considerations of harm to others. Instead, the case for compulsory vaccination needs to be strengthened by considerations of fairness. I have argued that vaccine refusal (a) is morally equivalent to tax evasion and (b) should be legally treated like tax evasion. This means that non‐vaccination should be illegal, except in cases where there are medical reasons for not vaccinating, and at least as long as we reject the libertarian claim that compulsory taxation itself is not ethically justified.

## Notes

1 Paul Fine, ‘Herd immunity: History, theory, practice’, *Epidemiologic Reviews*, 15, 2 (1993): 265–302

2 According to some philosophical views of coercion, even offers of incentives can in certain cases be coercive (see e.g. Virginia Held, ‘Coercion and coercive offers’ in Pennock J. Roland and John W. Chapman (eds.) *Nomos XIV: Coercion* (Chicago: Aldine‐Atherton 192)), because the offer might be irresistible for certain people. For the purpose of the present discussion, we can leave the issue of which policies are and are not coercive aside, as long as we agree that both mandatory and compulsory vaccination are coercive, even if at different degrees.

3 Mark Navin and M.A. Largent, ‘Improving nonmedical vaccine exemption policies: three case studies’, *Public Health Ethics*, 10, 3 (2017): 225–234

4 Roland Pierik, ‘Mandatory vaccination: an unqualified defense’, *Journal of Applied Philosophy*, 35, 2 (2018): 381–398

5 Pierik op. cit., note 4. Jessica Flanigan. ‘A defense of compulsory vaccination’, *HEC Forum*, 26 (2014): 5–25; Ben Bambery, Michael Selgelid, Hannah Maslen, Andrew Pollard, Julian Savulescu. The case for mandatory flu vaccination. *American Journal of Bioethics,* 13, 9 (2013): 38–40

6 Flanigan op. cit.

7 Pierik op. cit.

8 Flanigan op. cit. p. 20

9 Pierik op. cit.

10 Angus Dawson, ‘Herd protection as a public good: vaccination and our obligations to others’. In Angus Dawson and Marcel Verweij (eds), *Ethics, Prevention, and Public Health* (Oxford: Clarendon Press 2007): 160–187, p. 171

11 Dawson op. cit., p. 172

12 Flanigan op. cit, p. 12, emphasis added

13 Derek Parfit. *Reasons and Persons* (New York: Oxford University Press 1984), p. 81

14 Jason Brennan, ‘A libertarian case for mandatory vaccination’, *Journal of Medical Ethics*, 44, (2018): 37–43

15 Charlie Blunden, ‘Libertarianism and collective action: is there a libertarian case for mandatory vaccination?’ *Journal of Medical Ethics,* 45 (2018): 71–74

16 John Stuart Mill. *On Liberty*, (London: Walter Scott Publishing 1859), The Project Gutenberg Ebook of On Liberty, 2011 available at https://www.gutenberg.org/files/34901/34901-h/34901-h.htm , p. 17

17 Mill op. cit., p. 20

18 Mill op. cit., pp.140–141

19 George Klosko, *The Principle of Fairness and Political Obligation*, 2^nd^ edition (Lanham, MD: Rowman & Littlefield 2004 [1992])

20 Aaron James. *Fairness in Practice: a Social Contract for a Global Economy* (New York: Oxford University Press 2012) chapter 4

21 Dawson op. cit.

22 Mark Navin, *Values and Vaccine Refusal: Hard Questions in Ethics, Epistemology, and Health Care* (New York: Routledge 2015), p. 25; H Feldbaum et al. ‘Global health and national security: the need for critical engagement’, *Medicine, conflict and survival* 22, 3 (2006):192–198; UN Developing Program, *Human Development Report*: *New Dimensions of Human Security*. (New York, Oxford University Press 1994)

23 Fynnwin D. Wei Prager and A. Rose, ‘Total economic consequences of an influenza outbreak in the United States’, *Risk Analysis*. 37, 1 (2017): 4–19

24 Giubilini, A. , T. Douglas, J. Savulescu ‘The moral obligation to be vaccinated: utilitarianism, contractualism, and collective easy rescue’ *Medicine, Healthcare, and Philosophy*, 21, 4 (2018): 547–560

25 Giubilini, A. *The Ethics of Vaccination* (London: Palgrave 2019)

26 Jason Brennan, ‘Conscientious tax objection: why the liberal state must accommodate tax resisters’, *Public Affairs Quarterly*, 26, 2 (2012): 141–159

27 WHO, *Global Vaccine Safety. Six Common Misconceptions about Immunization*, 2018, at http://www.who.int/vaccine_safety/initiative/detection/immunization_misconceptions/en/index4.html , last access 7 August 2018

28 L. Al‐Ozaibi , Adnan J, Hassan B, Al‐Mazroui A, Al‐Badri F. ‘Seat belt syndrome: Delayed or missed intestinal injuries, a case reporroand review of literature’. *International Journal of Surgery Case Reports*, 20 (2016):74–76.

29 Giubilini, Douglas and Savulescu op. cit.

30 Navin 2015, op. cit. pp. 179–180

31 Mark Navin, ‘Resisting moral permissiveness about vaccine refusal’, *Public Affairs Quarterly*, 27, 1 (2013): 69–85; Navin M. 2015, op. cit. pp. 141–5; 155

32 Navin M. 2015, op. cit., p. 143.

33 Navin M. 2015, op. cit., p. 12

34 Gerald Dworkin, ‘Paternalism’, *The Stanford Encyclopedia of Philosophy *(Edward N. Zalta (ed.)), 2002 URL = <https://plato.stanford.edu/archives/fall2019/entries/paternalism/>.

35 Oxford Vaccine Group, Measles, 2017, at http://vk.ovg.ox.ac.uk/measles, last access 21 Dec 2017

36 CDC. Measles, mumps, and rubella (MMR) vaccine safety, 2017 at https://www.cdc.gov/vaccinesafety/vaccines/mmr-vaccine.html, last access 21 Dec 2017

37 M. Di Bonaventura, GB Chapman, ‘Do Decision Biases Predict Bad Decisions? Omission Bias, Naturalness Bias, and Influenza Vaccination’. *Medical Decision Making*. 28, 4 (2008): 532–539; Ilana Ritov and J Baron, ‘Reluctance to vaccinate. Omission bias and ambiguity’. *Journal of Behavioural Decision Making*, 3 (1992): 263–277

38 Giubilini 2019, op. cit. chapter 2

39 M. Mello, ‘Rationalizing vaccine injury compensation’. *Bioethics*. 22, 1 (2008): 32–42

40 C. Looker and H. Kelly, ‘No‐fault compensation following adverse events attributed to vaccination: a review of international programmes’, *Bulletin of the World Health Organization*, *89*, 5 (2011), 371–378

41 Dawson 2007 op cit. p. 176

42 Brennan 2012 op. cit

43 Ibidem, p. 249

44 Steve Clarke, A. Giubilini and M. Walker. ‘Conscientious objection to vaccination’, *Bioethics*, 31, 3 (2017): 155–161; Giubilini, A., T. Douglas, J. Savulescu, ‘Liberty, fairness, and the ‘contribution model’ for non‐medical vaccine exemption policies: A reply to Navin and Largent’, *Public Health Ethics*, 10, 3 (2017): 235–240

45 I am grateful to Mark Navin for having raised this objection during a private conversation.

46 Clarke, Giubilini, Walker op. cit.

47 Giubilini A. and J. Savulescu. ‘Demandingness and public health’, *Moral Philosophy and Politics*, 6, 1 (2019): 65–87

48
*Nature *553, 249–250 (2018)

49 D’Ancona, F. et al, ‘Introduction of new and reinforcement of existing compulsory vaccinations in Italy: first evaluation of the impact on vaccination coverage in 2017’. *Eurosurveillance*, 23, 22 (2018): pii=1800238

50 James F Childress, J. F., et al (2002). ‘Public health ethics: mapping the terrain’, *The Journal of Law, Medicine & Ethics*, 30, 2 (2002), 170–178; p. 173; Lawrence Gostin, *Public Health Law: Power, Duty, Restraint*. Revised and Expanded Second Edition (London: University of California Press 2008)

51 Verweij, Marcel and A. Dawson. ‘Ethical principles for collective immunization programs’, *Vaccine* 22 (2004): 3122–26; Navin 2015, op. cit., p. 162)

52 Pierik 2018, op. cit.

53 I am grateful to an anonymous reviewer for this observation.

54 Harris, John. ‘The survival lottery’, *Philosophy*, 50, 191 (1975): 81–87

